# Supplementary material for: Nutritional Quality, Environmental Impact and Cost of Ultra-Processed Foods: A UK Food-Based Analysis
Source: Int J Environ Res Public Health. 2022 Mar 8;19(6):3191. doi: 10.3390/ijerph19063191 (PMC8948822; doi:10.3390/ijerph19063191)
Supplement: Supplementary file 1 [file ijerph-19-03191-s001.zip › ijerph-1580267-supplementary.pdf]

International Journal of Environmental Research and Public Health

MDPI

Supplementary Material

Table S1. Shapiro–Wilk test values among dimensions studied per 100 kcal.

Category	NRF9.3	GHGE	Cost
All items	W = 0.99524, p-value = 1.814 x 10^{-11}	W = 0.54645, p-value < 2.2 x 10^{-16}	W = 0.20872, p-value < 2.2 x 10^{-16}
Fruit and vegetables	W = 0.9571, p-value = 1.555 x 10^{-14}	W = 0.54036, p-value < 2.2 x 10^{-16}	W = 0.61444, p-value < 2.2 x 10^{-16}
Potatoes, bread, rice, pasta and other starchy carbohydrates	W = 0.9896, p-value = 3.286 x 10^{-08}	W = 0.64448, p-value < 2.2 x 10^{-16}	W = 0.35481, p-value < 2.2 x 10^{-16}
Beans, pulses, fish, eggs, meat and other proteins	W = 0.9812, p-value = 4.539 x 10^{-14}	W = 0.7607, p-value < 2.2 x 10^{-16}	W = 0.28451, p-value < 2.2 x 10^{-16}
Dairy and alternatives	W = 0.99099, p-value = 0.0227	W = 0.68748, p-value < 2.2 x 10^{-16}	W = 0.22126, p-value < 2.2 x 10^{-16}
Drinks	W = 0.98589, p-value = 0.02606	W = 0.5513, p-value < 2.2 x 10^{-16}	W = 0.41501, p-value < 2.2 x 10^{-16}
Items that should be eaten less often and in small amounts	W = 0.97388, p-value = 2.162 x 10^{-05}	W = 0.70617, p-value < 2.2 x 10^{-16}	W = 0.15579, p-value < 2.2 x 10^{-16}

Since all the p-value were <0.05, indicating significantly non-normality. Hence non-parametric tests were selected. NRF8.3 = Nutrient-Rich Food Index 8.3, GHGE = Green House Gas Emission.

Table S2. Overall median values across dimensions and categories.

Category	NRF8.3	GHGE	Cost
All items	300	418	0.5
Fruit and vegetables	120	210	0.35
Potatoes, bread, rice, pasta, and other starchy carbohydrates	260	651	0.25
Beans, pulses, fish, eggs, meat, and other proteins	460	470	0.62
Dairy and alternatives	158	193	0.25
Drinks	72	42	0.26
High fat, salt, or sugar products	400	468	0.58

NRF8.3 = Nutrient-Rich Food Index 8.3, GHGE = Green House Gas Emission.
